# A Synergy of Convolutional Neural Networks for Sensor-Based EEG Brain–Computer Interfaces to Enhance Motor Imagery Classification

**DOI:** 10.3390/s25020443

**Published:** 2025-01-13

**Authors:** Souheyl Mallat, Emna Hkiri, Abdullah M. Albarrak, Borhen Louhichi

**Affiliations:** 1 Department of Computer Science, Faculty of Sciences, Monastir University, Monastir 5019, Tunisia; souheyl.mallat@gmail.com; 2 Department of Computer Science, Higher Institute of Computer Science, Kairouan University, Kairouan 3100, Tunisia; emna.hkiri@gmail.com; 3 Department of Computer Science, College of Computer and Information Sciences, Imam Mohammad Ibn Saud Islamic University, Riyadh 11432, Saudi Arabia; 4 Department of Mechanical Engineering, College of Engineering, Imam Mohammad Ibn Saud Islamic University, Riyadh 11432, Saudi Arabia; blouhichi@imamu.edu.sa

**Keywords:** brain–computer interface, electroencephalography, deep learning, convolutional neural network

## Abstract

Enhancing motor disability assessment and its imagery classification is a significant concern in contemporary medical practice, necessitating reliable solutions to improve patient outcomes. One promising avenue is the use of brain–computer interfaces (BCIs), which establish a direct communication pathway between users and machines. This technology holds the potential to revolutionize human–machine interaction, especially for individuals diagnosed with motor disabilities. Despite this promise, extracting reliable control signals from noisy brain data remains a critical challenge. In this paper, we introduce a novel approach leveraging the collaborative synergy of five convolutional neural network (CNN) models to improve the classification accuracy of motor imagery tasks, which are essential components of BCI systems. Our method demonstrates exceptional performance, achieving an accuracy of 79.44% on the BCI Competition IV 2a dataset, surpassing existing state-of-the-art techniques in using multiple CNN models. This advancement offers significant promise for enhancing the efficacy and versatility of BCIs in a wide range of real-world applications, from assistive technologies to neurorehabilitation, thereby providing robust solutions for individuals with motor disabilities.

## 1. Introduction

### 1.1. Overview

Brain–computer interfaces (BCIs) represent a groundbreaking technology for diagnosing motor disabilities and facilitating direct communication between the human brain and machines, revolutionizing human–machine interaction [1]. Among their myriad applications, BCIs hold particular promise for empowering individuals with disabilities and augmenting assistive technologies. A fundamental challenge in BCI research lies in extracting robust control signals from inherently noisy brain data, a task exacerbated in motor imagery tasks [2]. Motor imagery, where users mentally simulate specific movements, is a pivotal component of BCI systems, enabling control over prosthetics, exoskeletons, and other assistive devices.

Despite significant strides in BCI technology, the quest for reliable control signals remains fraught with obstacles, ranging from limited signal resolution to inter-subject variability. Previous studies have reported classification accuracies for motor imagery tasks ranging from 60% to 90%, underscoring the imperative for further advancements to bolster the usability and dependability of BCI systems [3,4,5]. Addressing this challenge, our paper presents a novel method leveraging convolutional neural networks (CNNs) to enhance the classification performance of motor imagery in BCI applications. CNNs have emerged as powerful tools for extracting intricate features from complex data, making them well suited for analyzing brain signals.

### 1.2. Background

These neural network systems leverage the benefits of various modalities to improve BCI performance after analyzing the situation of motor disability. The advantages of hybrid BCIs over single-mode BCIs, in terms of increased accuracy and efficiency, have been documented in a number of studies. In recent times, numerous research papers have been put forward to explore the application of deep learning techniques, such as CNN, long short-term memory (LSTM), and restricted Boltzmann machines (RBM), for the purpose of automatic feature extraction and categorization. The outcomes of these methodologies have demonstrated that they diminish the time-consuming preprocessing and achieve a superior level of accuracy [1,2]. Deep learning models have drawn a lot of interest in the field of neural signal processing and decoding due to recent developments in the field and its extensive applications in computer vision and natural language processing.

### 1.3. Contribution and Scope

In recent years, there has been burgeoning interest in leveraging electroencephalography (EEG) brain signals for a myriad of applications, particularly in the healthcare domain. BCIs offer an alternative avenue for communication between humans and machines, circumventing the conventional pathways of nerves and muscles. By recording, analyzing, and translating the user’s brain activity into actionable commands, BCIs enable individuals to express their intentions without physical movement. Motor imagery, in this context, has garnered significant attention as a means to diagnose motor dysfunctions stemming from diseases, limb impairments, or spinal nerve injuries, provided that brain function remains intact and the user is conscious.

Main objectives of the study are as follows:

- We aim to leverage a fusion of convolutional neural network methods to refine the classification of motor imagery EEG (MI-EEG) signals, thereby contributing to the advancement of BCI technology.
- In this study, we focus on a specific dataset, the BCI competition IV 2a dataset of nine persons from the EEG dataset, specifically addressing tasks associated with motor imagery actions.
- To propose and evaluate a convolutional neural network-based framework for MI-EEG signal classification, aiming to outperform existing methodologies and advance the state-of-the-art in BCI technology.

### 1.4. Organization of the Paper

This paper unfolds as follows: In Section 2, we provide a comprehensive review of related work, elucidating the existing landscape and identifying gaps. Section 3 delves into the dataset utilized and offers a detailed exposition of our proposed methodology. Subsequently, in Section 4, we meticulously analyze the experimental results to substantiate the efficacy of our proposed method. Finally, Section 5 encapsulates our findings and outlines avenues for future research.

## 2. Materials and Methods

### 2.1. Related Work

The cornerstone of a BCI system resides in its capacity to extract EEG features to enhance recognition accuracy, a pivotal component for overall system performance [1]. Initially, machine learning methodologies such as linear discriminant analysis (LDA) [2], the support vector machine (SVM) classification algorithm [3], k-nearest neighbor (KNN) [4], and naïve Bayes (NB) [5,6,7,8,9,10] displayed inadequate performance. These approaches hinge on simplifying assumptions that may not always hold true for EEG signals. For instance, LDA presupposes data linearity, a rarity in EEG data.

SVMs [3] offer a remedy to practical challenges such as small sample sizes, non-linear relationships, high dimensions, and local minima, resulting in more accurate classification. However, the selection of kernel functions remains intricate in real-world applications due to the random and non-stationary nature of EEG signals, coupled with a lack of prior knowledge regarding signal distribution characteristics. In [7], authors substituted the support vector machine (SVM) classifier with the multilayer perceptron (MLP).

Furthermore, a fuzzy logic-based approach is referenced. Specifically, Fabien et al. [8] extracted power features from C3 and C4 electrodes in the alpha and beta bands to generate feature sets utilized as inputs for a fuzzy system tailored for two-class (binary) MI classification. Xu et al. [10] employed the wavelet transform (WT) to produce feature sets based on a three-level decomposition tree structure, selecting sub-bands corresponding to µ and β rhythms from C3 and C4 channels. These extracted feature sets were integrated into a binary fuzzy SVM classifier utilizing a radial basis function kernel.

All the aforementioned works were confined to binary classification problems. To bridge this gap, a solution for multiple-class MI data classification is proposed, founded on a multioutput fuzzy logic system (FLS) [9,10]. This fuzzy system is formulated by amalgamating with the particle swarm optimization (PSO) method, a population-based metaheuristic, to enhance classification performance. Several competing approaches are deployed for comparison with the multi-class fuzzy system based on PSO. The computational complexity of the FLS learning process poses limitations to this approach, which could be mitigated by exploring more efficient optimization strategies. Moreover, feature extraction and selection could be refined by increasing the number of extracted features and exploring different feature selection methods. Evaluating alternative feature extraction techniques could potentially enhance common spatial patterns (CSPs) performance.

Various other machine learning-based methods [11] have been devised for decoding imagined movements and feature extraction. Among these, the common spatial filter bank (FBCSP) model, predicated on the CSP method, has demonstrated slightly superior performance. Numerous researchers have delved into the potential of deep learning models such as common Bayesian network (CBN) [6], LSTM [12], recurrent neural network (RNN) [13], deep belief networks (DBN) [14], and stacked autoencoder (SAE) [15] for EEG signal analysis, striving to extract more robust and discriminative features. However, the application of CNN and other deep learning models to EEG MI data has yet to yield significant improvements compared to traditional machine learning techniques, indicating the need for further advancements.

Recently, attention has shifted towards feature fusion from different models, a promising avenue to enhance classification performance. For instance, researchers in [15] proposed a method combining ConvNet with MLP within a deep learning framework to create another classifier for multi-class imaginary motor task classification. Similarly, the work by Xu et al. [16] introduced an approach combining CNN with the gradient boosting (GB) algorithm, illustrating the trend of merging deep learning with machine learning approaches to extract meaningful information from EEG signals.

In the same vein, Sakhavi et al. [17] introduced four models, namely CNN, CNN+LSTM, CNN-SVM, and CNN+LSTMSVM, utilizing the BCI Competition IV 2a dataset. Experimental results showcased that the proposed model, CNN-SVM, attained a performance of approximately 64%. In Zhao et al. [18], a method combining multibranch 3D CNN and 3D representation of EEG is presented, extracting temporal and spatial features simultaneously, thereby fully exploiting their relationship. Other researchers explore the utilization of CNN in combination with FBCSP [19,20] and other methodologies to extract temporal features.

Moreover, the intricacies of deep learning techniques such as ConvNet, DBN [6], and restricted Boltzmann networks (RBNs) [21] in decoding EEG signals based on mental activities have been recently demonstrated and widely utilized for analyzing distinctive features and categorizing EEG signals. For instance, certain studies amalgamated multiple RBNs to extract features from imagined movements. Additionally, other researchers explored the joint utilization of ConvNet and RNN [22] to extract spatiotemporal features and multidimensional features, identifying cognitive events from EEG signals. In a particular study, ConvNet features and CSP [23] were amalgamated to form representative features. Another study employed ConvNet and autoencoders to recognize emotions from EEG signals.

Regarding fusion, various approaches are explored, involving the combination of n models of multilayer CNNs, each with a specific configuration of parameters [24]. For instance, the study conducted by [25] introduced a fusion method of MCNN and CCNN that leverages different convolution features to capture spatial and temporal aspects of raw EEG data. This fusion method, presented in this research, outperforms all the most advanced machine learning and deep learning techniques in the EEG classification field. Various experiments were conducted to evaluate the performance of the CNN fusion method on public datasets.

The results demonstrate that this method [24,25] achieves remarkable performances of 75.7% and 95.4% on the BCI Competition IV-2a dataset and the High Gamma dataset, respectively. This method necessitates more data and computational resources for training. Additionally, it requires large amounts of training data, which can be a hurdle for clinical applications. Finally, it may be sensitive to noise in EEG signals, leading to decreased classification accuracy. Feature fusion and different CNN architectures have not been thoroughly explored and exploited for EEG data currently. Further, Table 1 shows the comparative analysis of various methodologies and their performance in EEG signal analysis.

Table 1 provides a comparative overview of various methodologies employed in EEG signal analysis, highlighting their descriptions and performance outcomes. Drawing inspiration from recent studies, particularly one investigating multilayer CNN fusion models in EEG analysis, our proposed work delves into this domain. Using task-specific knowledge to create the network architecture, these studies in the literature mostly concentrated on categorization in a particular BCI task.

Moreover, owing in part to the challenges in gathering data under various experimental designs, the quantity of data utilized to train these networks differed substantially across research. Therefore, it is still not apparent how well these earlier neural network learning methods will adapt to different types of BCI tasks and different quantities of training data.

Specifically, we endeavor to explore the fusion of distinct CNN models, each distinguished by unique characteristics such as the number of convolution blocks, activation functions, max pooling operations, and a Flatten layer. This methodology seeks to leverage the strengths of diverse CNN architectures to enhance the classification performance of EEG signals. By integrating insights from various CNN configurations, we aim to devise a robust model by hybridizing five CNNs for EEG data, thereby advancing the efficacy of EEG-based BCI classification systems.

### 2.2. Methodology

In this section, we will delve into the dataset utilized in this research study. Following that, we will present a detailed description of the proposed approach for classifying imagined movements. Our methodology involves the fusion of multiple CNN models to enhance classification accuracy.

#### 2.2.1. Structure of the CNN Fusion Model 

Convolutional neural networks (CNNs), a subset of deep feed-forward neural networks, have shown remarkable potential in various tasks, such as image classification, object detection, and segmentation. The architecture of a CNN exhibits unique characteristics compared to other neural network types, such as multi-layer perceptrons. CNN layers are structured in three dimensions: width, height, and depth. Neurons within a single layer connect exclusively to a subset of neurons in the subsequent layer rather than to all neurons present.

A basic CNN architecture consists of multiple layers, where each layer transforms one activation volume into another using a differentiable function [28]. The primary types of layers used in constructing CNN architectures include the convolutional layer, the ReLU layer, the pooling layer, and the fully connected layer.

Unlike individual machine learning models, fusion models leverage the complementary advantages of multiple models to achieve superior performance and reduced error. The foundational learners employed in this study were subsequently combined using a stacking strategy. Support vector machine, GrowNET, random forest, XGBoost, and LightGBM were the enhanced algorithms utilized, all derived from the gradient boosting decision tree (GBDT) algorithm. These models exhibit characteristics such as robustness to input requirements, low computational complexity, and effective predictive performance.

**Figure 1:**
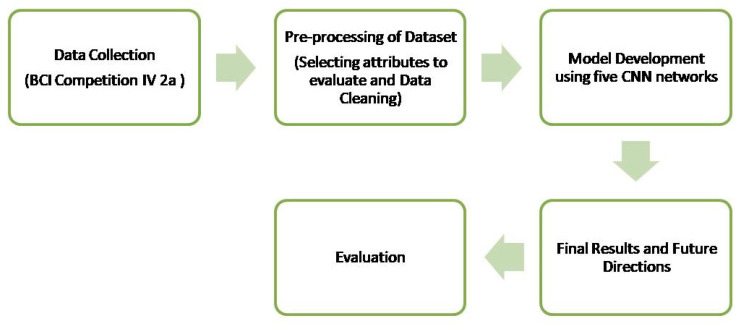
The flowchart of the methodology.

Nevertheless, each of the five models demonstrates distinct advantages in different contexts. For example, random forests are more interpretable than gradient boosting trees (GBTs). The methodology flow is illustrated in Figure 1 with a diagram.

#### 2.2.2. Dataset Description

**Figure 2:**
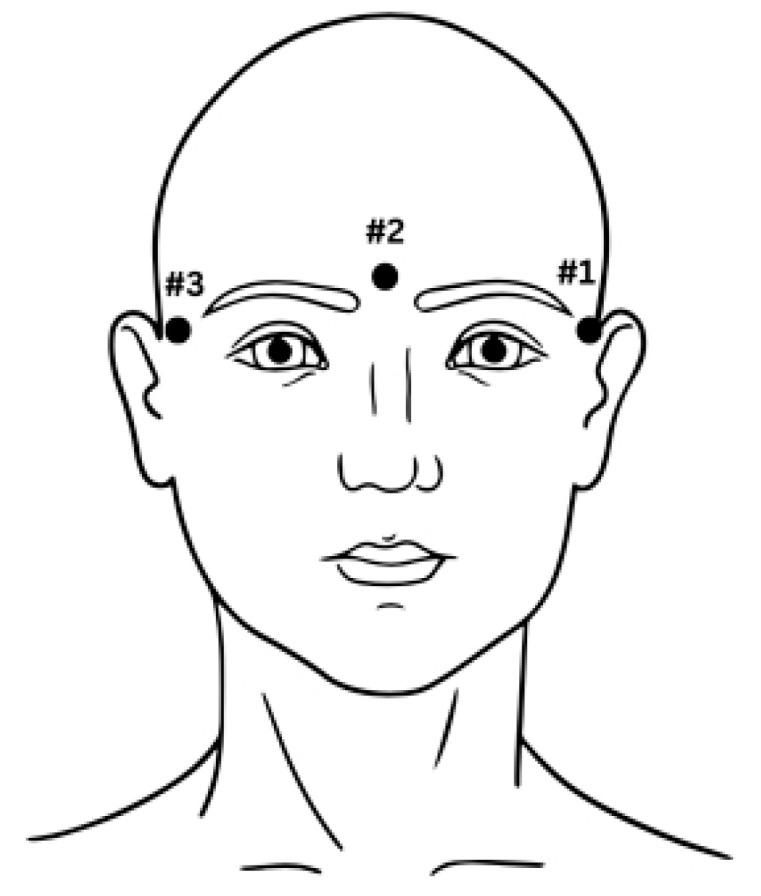
Positioning of the three EOG electrodes.

In this study, we utilized the BCI Competition IV 2a dataset to train and evaluate our proposed method, enabling a comparative analysis with other state-of-the-art techniques. BCI Competition IV 2a dataset consists of data from only nine subjects. The BCI Competition IV 2a dataset consists of EEG data collected from 9 subjects (5 males and 4 females) aged between 20 and 30 years. The subjects were healthy, with no prior history of neurological disorders. The data were recorded during a motor imagery task, with subjects imagining left-hand or right-hand movements while their brain activity was captured using 22 EEG channels. This dataset was designed for the evaluation of MI-based BCI algorithms. Each of the nine subjects performed four different motor imagery tasks: left-hand, right-hand, both feet, and tongue. The dataset includes EEG recordings with 22 electrodes, sampled at 250 Hz. These recordings are segmented into trials, with each trial comprising a baseline period and a motor imagery task period. In BCI research, a clear specification of the number of subjects and the details of the experimental setup are critical for reproducibility and benchmarking. In this work we have taken the dataset, which encompasses EEG signal recordings from 9 subjects who were comfortably seated and engaged in MI tasks. The recordings were captured using 22 EEG channels (denoted as Fz, FC3, FC1, FCz, FC2, FC4, C5, C3, C1, Cz, C2, C4, C6, CP3, CP1, CPz, CP2, CP4, P1, Pz, P2, POz) and 3 electrooculography (EOG) channels. The electrodes were positioned on the scalp according to the standard 10–20 system, as illustrated in Brunner et al. [25] and in Figure 2.

**Figure 3:**
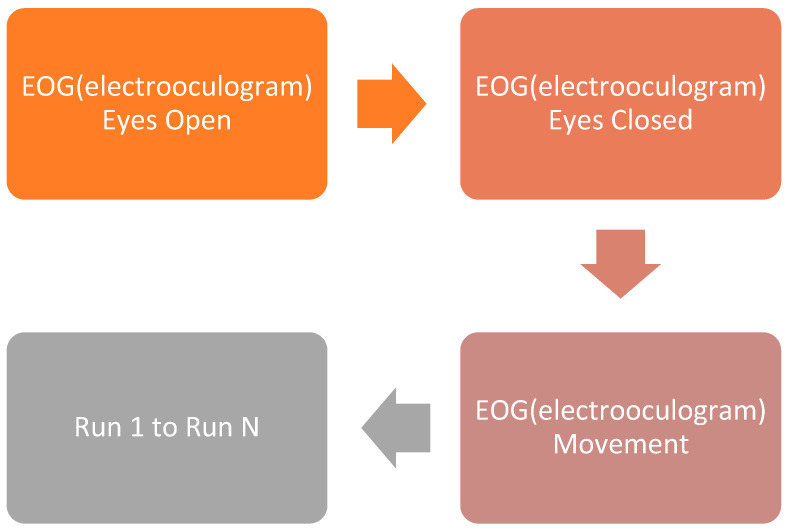
Schedule of a session.

Brunner et al. [25] show the placement of 22 EEG electrodes taken for the study. In Figure 3, positioning of three EOG electrodes is shown on the subject. The subjects performed four distinct MI tasks representing movements of the left-hand, tongue, feet, and right-hand. The data were segmented into short series, each comprising 48 trials of each MI activity. Data collection spanned two sessions conducted over two days, with each session consisting of six runs interspersed with brief breaks. Consequently, a total of 288 trials were collected for each motor imagery activity. Each session commenced with approximately 5 min of EEG data recording to estimate the influence of the 3 EOG channels.

The earliest attempts to overcome inter-session variability involved preliminary training sessions aimed at enhancing the user’s ability to modulate brain signals robustly enough to control BCIs. However, these training sessions were often tedious and inconvenient for users. To address this, machine learning-based BCI models were introduced, reducing the need for individual training sessions for each BCI application. In these models, calibration is performed using data collected at the beginning of each session. Inter-subject variability in brain topography, a fundamental feature of the brain, arises due to several factors:

- *Molecular biology:* both genetic and environmental factors play significant roles in shaping an individual’s brain;
- *Mental approach:* variability can stem from differences in how individuals approach and think about tasks;
- *Structural brain features:* cortical thickness and the number of folds in the brain are correlated with the functioning of neural networks across the brain;
- *Cognitive variables:* factors such as attention, fatigue, and reaction time, which change over time, can modulate brain activity and performance.

Interpreting and deciphering brain activity is challenging due to inter-subject heterogeneity. However, researchers have embraced this diversity to gain deeper insights into human variation and brain function. Some approaches to address these challenges include:

- *Transfer Learning:* By accounting for differences in data across subjects, transfer learning can enhance brain decoding performance. This technique aims to identify a learning model that remains consistent across multiple subjects or sessions, referred to as invariant representation.
- *Domain Adaptation:* This is one of the most commonly used techniques in implementing BCIs.

Figure 3 illustrates the montage mapping of EEG and EOG channels in panels (a) and (b), respectively. The recording process is divided into three blocks as follows:

- Subjects spend two minutes with eyes open, fixating on a static cross displayed on the screen.
- Following the period with eyes open, participants undergo one minute of eyes-closed resting.
- Subsequently, for one minute, participants engage in controlled eye movements, as illustrated in Figure 3.

Figure 3 shows the schedule of the session performed with all the subjects. First, the data were taken with eyes open, and then eyes-closed data were taken. After that run was performed on the data. Due to technical issues in the IV2a dataset, the EOG block is shorter for subject 4 and only includes the eye movement condition. The timing of data acquisition is depicted in Brunner et al. [25]. It is shown that at the beginning of each trial (t = 0 s), a fixation cross appears on the black screen, accompanied by a brief audible warning tone. After two seconds (t = 2 s), an arrow indicating left, right, down, or up (corresponding to one of the 4 classes) appears and remains on the screen for 1.25 s. This prompts the subjects to perform the desired MI task until the fixation cross disappears from the screen at t = 6 s. Finally, a brief pause with a black screen is introduced between trials, which is shown in Brunner et al. [25]. Specifically, the EEG signals were segmented using a sliding window approach with a window size of 2 s and an overlap of 50%. This segmentation is crucial for capturing temporal dynamics and improving feature extraction. Our CNN model comprises three convolutional layers with their respective kernel sizes, followed by max-pooling layers and two fully connected layers. For hyperparameter tuning, we employed grid search methodology, adjusting learning rates (ranging from 0.001 to 0.01), batch sizes (16, 32, and 64), and dropout rates (0.3 to 0.5). This fine-tuning was validated using cross-validation on the training set to ensure optimal performance.

#### 2.2.3. Proposed Approach

**Figure 4:**
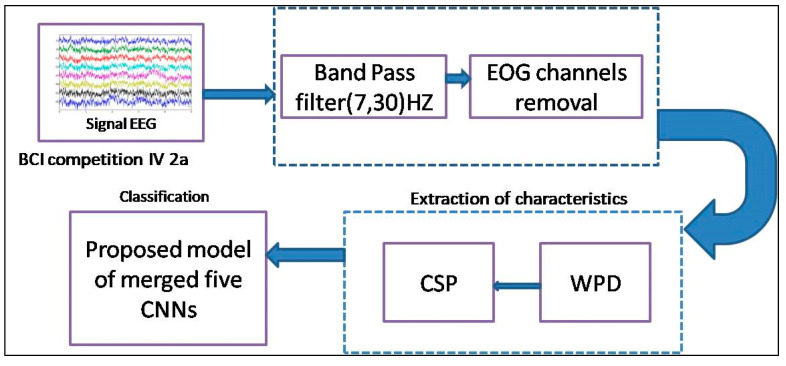
General diagram of our proposed motor imagery classification method.

**Figure 5:**
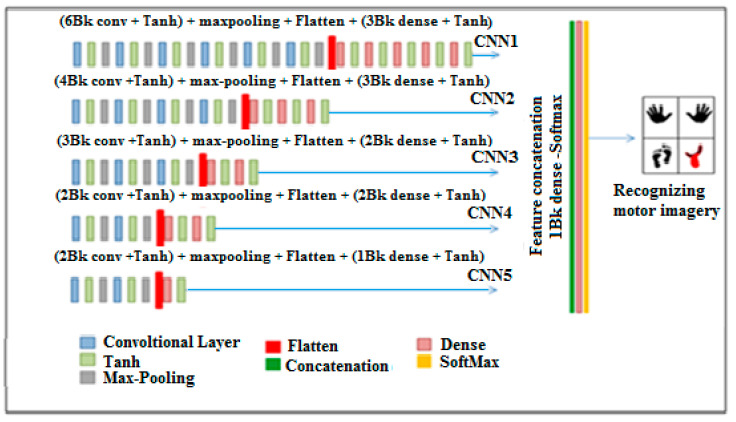
Classification through merger of the five CNN models.

Figure 4 illustrates the architecture of our system, showcasing the implementation of our proposed method. The process commences with a streamlined preprocessing of EEG signals, entailing the application of a 7 to 30 Hz band-pass filter and the exclusion of the 3 EOG channels, thus preserving solely the 22 EEG channels. Subsequently, we employ two distinct models: wavelet packet decomposition (WPD) for discerning frequency characteristics and common spatial pattern (CSP) for delineating spatial characteristics. These extracted frequency and spatial characteristics serve as inputs for our proposed model. The common definition and mechanism used in the proposed approach are outlined as follows:

- *Indexing and Ranking Mechanism:* Wavelet packet decomposition (WPD) is a variant of wavelet transform employed for analyzing EEG signals, wherein the signals undergo successive transformations via discrete-time filters, resulting in discrete wavelets. Unlike the traditional discrete wavelet transform (DWT), where each level is determined solely by the approximation coefficients of the preceding wavelet, WPD involves decomposing both the approximation and detail coefficients into multiple levels, generating a binary tree structure. This expanded decomposition in WPD offers enhanced frequency resolution compared to standard wavelet analysis, as it allows for a finer-grained analysis of both approximation and detail coefficients at each level. Consequently, wavelet packet analysis (WPA) emerges as a more precise method for dissecting EEG signals, offering valuable insights into their frequency characteristics.
- *Wavelet Packet Decomposition (WPD):* Coifman and Wickerhauser [29] pioneered the concept of wavelet packets, introducing a more intricate decomposition method for signals based on orthogonal wavelets. By employing orthogonal wavelets, they proposed orthogonal wavelet packets, which facilitate detailed decomposition of both low-frequency and high-frequency signal components. Unlike the wavelet transform, this decomposition offers neither redundancy nor omission, thereby enhancing the signal’s time-frequency localization analysis capacity, particularly for vibrational signals containing medium and high-frequency information. This advancement aims to improve the accuracy of EEG recognition and can be viewed as a spatial decomposition technique.
- *Common Spatial Pattern (CSP):* The common spatial pattern (CSP) algorithm serves as a feature extraction technique rooted in the theory of diagonalization of the covariance matrix within a two-class signal framework. Fundamentally, CSP aims to identify the optimal projection matrix that maximizes the variance for one class while simultaneously minimizing it for the other. This strategic approach yields maximum contrast between the two classes, facilitating robust discrimination, such as between right-hand and left-hand movements. As a spatial filtering method, CSP effectively enhances the distinction between target classes. Widely adopted in brain–machine interface systems, the CSP algorithm plays a pivotal role in extracting motor imagery features, contributing to advancements in neurotechnology [16,30].
- *Merging of the five CNNs:* In our proposed approach, we employ five CNNs to discern motor imagery patterns. This involves amalgamating the outputs of these five CNN models through a straightforward concatenation process, followed by the application of a dense layer equipped with an activation function. The five CNNs were chosen based on a combination of their architectural diversity, performance benchmarks in related applications, and compatibility with our dataset. Each CNN model contributes unique characteristics, such as varying filter sizes, depth, and activation mechanisms, which collectively enhance the merged architecture. This diversity allows for better feature extraction and minimizes the risk of overfitting to specific patterns in the data. Additionally, these models have demonstrated high classification accuracy and robustness in similar domains during our preliminary investigations.
- The overarching architecture of our proposed methodology is depicted in Figure 5, providing a visual representation of the model’s structure and workflow.

The specifications of the five CNN models utilized in this study are outlined as follows: CNN 1 comprises 6 convolution blocks with Tanh activation and max pooling, succeeded by a Flatten layer. Subsequently, it incorporates 3 blocks, each housing a dense layer with Tanh activation. Similarly, CNN 2 consists of 4 convolution blocks with Tanh activation and max pooling, followed by a Flatten layer, and then 3 blocks, each featuring a dense layer with Tanh activation. Moving on to CNN 3, it encompasses 3 convolution blocks with Tanh activation and max pooling, succeeded by a Flatten layer. It further integrates 2 blocks, each comprising a dense layer with Tanh activation. CNN 4 follows a similar pattern with 2 convolution blocks, Tanh activation, and max pooling, followed by a Flatten layer, and then 2 blocks, each housing a dense layer with Tanh activation. Lastly, CNN 5 includes 2 convolution blocks with Tanh activation and max pooling, followed by a Flatten layer, and then a block containing a dense layer with Tanh activation. The merging of these CNNs involves a straightforward concatenation process followed by a dense layer with the “SoftMax” activation function. There are different ways to mitigate noise during classification, some of which are used in this study are as follows:

- *Preprocessing Techniques:* We utilize advanced filtering techniques, including band-pass filters, to remove high-frequency noise and artifacts, which are common in EEG data. This preprocessing step ensures that only the relevant frequency bands are considered for classification.
- *Noise Robustness through Feature Extraction:* To further reduce the impact of noise, we have employed feature extraction methods that are inherently more robust to noise, such as wavelet transforms and time-frequency domain features. These techniques help preserve the underlying patterns in EEG signals while minimizing the effect of noise.
- *Data Augmentation:* To improve the model’s ability to generalize in noisy environments, we have introduced data augmentation strategies, such as adding synthetic noise during training. This allows the model to better handle variations in real-world EEG data and improves its robustness against noise.
- *Regularization Methods:* We have also incorporated regularization techniques, including dropout and weight decay, to prevent overfitting to noisy data, thus enhancing the model’s performance and stability when dealing with noisy EEG signals. The results achieved after the analysis are described in the next section.

## 3. Data Analysis and Results

In this section, we delve into the main results derived from the amalgamation of the proposed CNNs. As is evident, deep learning (DL) necessitates substantial computational resources. A 3 × 3 window with a stride of 1 was chosen. Hyperparameters of CNNs are as follows:

- *Kernel Size:* We have specified the kernel size as used in our models, which are [3,4,6,8,15].
- *Number of Layers:* Number of layers for each model, including the convolutional, pooling, and fully connected layers, is two fully connected layers, and each utilizing 32 neurons.
- *Pooling Methods:* We have used max pooling and local average pooling methods.

A total of 95% memory usage training, even for a simple deep learning model on a conventional laptop, can consume hours, if not days. Efficient operation of DL often mandates a robust system. Thankfully, the utilization of GPUs and TPUs can significantly truncate training durations, completing tasks in mere minutes or seconds. However, the cost associated with acquiring GPUs can be prohibitive for many individuals. This is where Google Colab emerges as a pivotal solution. Google Colab stands as a complimentary cloud service provided by Google, meticulously crafted to streamline research endeavors in machine learning and artificial intelligence. It furnishes a potent platform for expedited learning and development of deep learning models, all within the familiar confines of the Python programming language.

### 3.1. Proposed System Results

Performance metrics are quantitative measures used to evaluate the efficacy and quality of a model, algorithm, or system in achieving its intended outcomes. Common metrics include accuracy, precision, recall, F1 score, and area under the curve (AUC) for receiver operating characteristic (ROC) curves. The performance metrics considered here include robustness and efficiency. Table 2 presents the outcomes of combining the five CNN models, which yielded a mean precision value of 80.19% for classification. Notably, Subject 04 exhibited a lower precision value of 44.83%, in contrast to Subject 03, which achieved the highest precision value of 84.48%. This discrepancy suggests that the EEG signals of Subject 04 were less distinct and more susceptible to noise, posing a greater challenge for accurate classification by the models.

**Figure 6:**
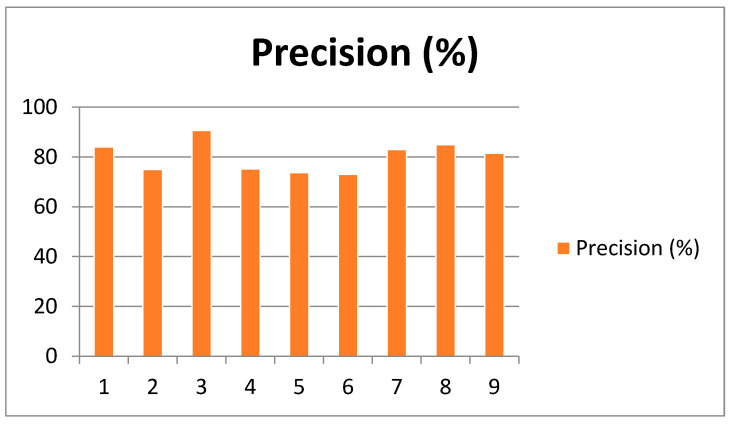
Graphical representation of precision achieved for Subjects 1 through 9.

The EOG block is designed to detect eye movements, which can disrupt EEG signals. If the EOG block duration is shorter for Subject 04, the CNN models may face greater difficulty in identifying and mitigating the impact of eye movements, potentially resulting in reduced classification accuracy. Table 2 and Figure 6 show the precision results achieved for Subjects 1 to 9, respectively.

Table 3, Table 4, Table 5, Table 6, Table 7, Table 8, Table 9, Table 10 and Table 11 present the precision, recall, and F1 score of our proposed methods across all nine subjects for the identification of four distinct motor imagery tasks: left-hand, right-hand, feet, and tongue. Notably, our analysis reveals that among the five models examined, the highest classification precision of 91% was achieved for Subject 7 in the feet class. Furthermore, Subjects 8 and 9 demonstrated exceptional recall rates of 100% for the tongue class and the feet class, respectively. Additionally, Subject 9 attained the highest F1 score of 91.3% in the feet class, underscoring the robustness and effectiveness of our proposed approach.

### 3.2. Accuracy vs. Epoch

**Figure 7:**
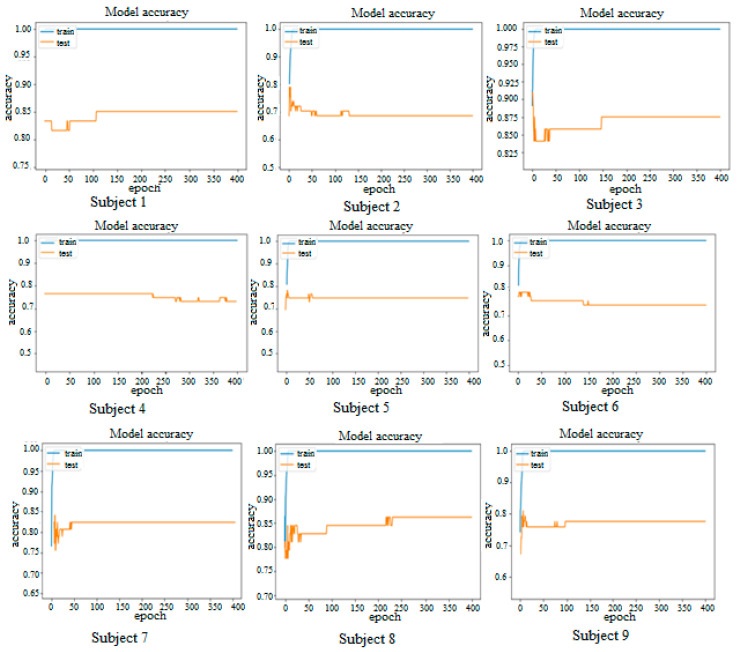
Accuracy vs. epoch for all nine subjects.

Figure 7 visually depicts the progression of accuracy in the motor imagery EEG signal classification model across epochs on both the training and test datasets. Notably, on the training set, accuracy exhibits a swift rise at the outset of training, surging to approximately 90% after 25 epochs. Subsequently, the accuracy stabilizes at this elevated level for the remainder of the training duration.

The accuracy trend on the test set follows a gradual increase at the onset of training, eventually reaching approximately 80% after 25 epochs. This upward trajectory persists throughout training, albeit at a slower pace, indicating ongoing potential for enhancement. The sustained rise in accuracy until the conclusion of training implies that the model may still benefit from further refinement, albeit with diminishing returns as training progresses.

Conversely, the accuracy curve for Subject 4 on the test set exhibits a distinct pattern. Initially, there is a gradual increase in accuracy during the early stages of training, culminating in a plateau around 65% after 25 epochs. This plateau persists until epoch 150, after which accuracy begins to fluctuate, oscillating between 60% and 70%. While the initial increase in accuracy suggests room for improvement, the subsequent fluctuation raises concerns regarding overfitting. This fluctuation may stem from the presence of weak and ambiguous motor imagery EEG signals, which pose a greater challenge for classification compared to stronger, clearer signals. Consequently, while the model demonstrates potential for improvement initially, the fluctuation post-epoch 150 underscores a heightened susceptibility to less distinct signals, potentially leading to erroneous interpretations of the data.

### 3.3. Loss vs. Epoch

**Figure 8:**
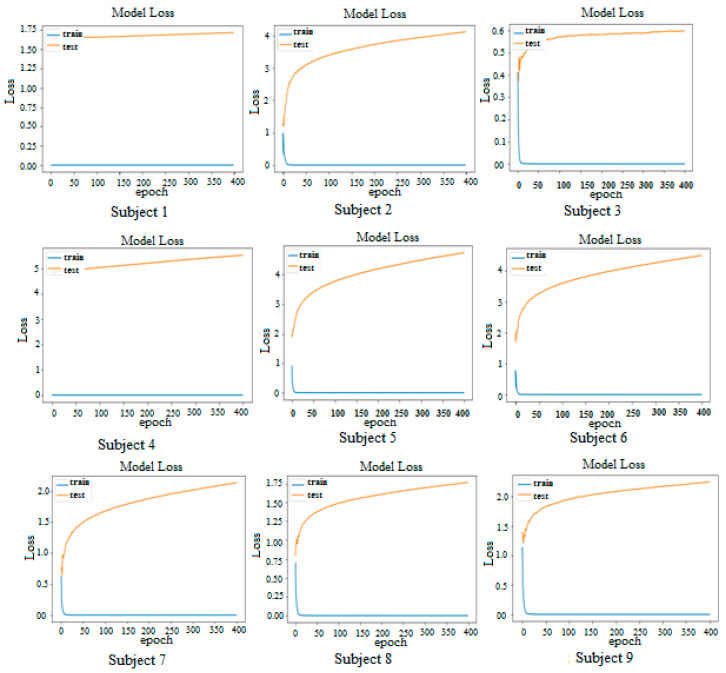
Loss vs. epoch for all nine subjects.

The examination of loss trends across epochs for the motor imagery EEG signal classification model yields noteworthy insights, as depicted in Figure 8.

Observing the growth of epochs from 1 to 25 reveals a notable increase in the accuracy rate on the training set, climbing from approximately 70% to 10% across all nine subjects before plateauing. However, the accuracy rate on the test set displays considerable variation up to around 150 epochs, followed by stabilization around 0.75 to 0.80 until 400 epochs for most subjects. Subsequently, there is a gradual increase and stabilization, indicating the model’s proficiency in classification. The analysis of the loss curve demonstrates a consistent downward trend for all subjects, indicative of the model’s learning phase and enhancement in accuracy. The low loss values at the final epoch (below 0.2 for all subjects) underscore the model’s adeptness in classifying EEG signals with precision. Furthermore, the convergence of the curve across all subjects signifies that the model has attained its peak accuracy, reaffirming the reliability of its classification capability.

Subject 4 stands as an exception, displaying a slight increase in loss at the final epoch compared to other subjects. The loss value at the final epoch for Subject 4 registers at 0.25, a significant value of nonetheless. However, this marginal rise in loss may be attributed to the lower quality of EEG signals from Subject 4 compared to those of other subjects.

### 3.4. Confusion Matrices

**Figure 9:**
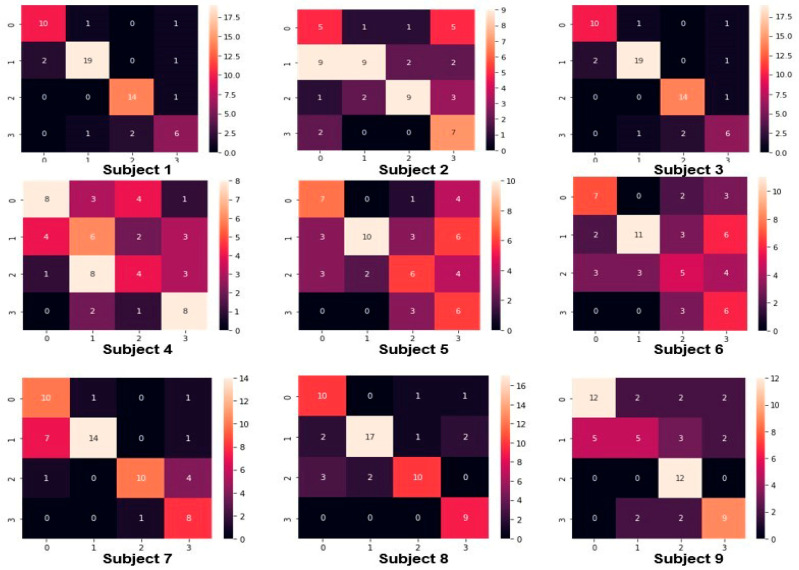
The confusion matrix obtained from motor imagery EEG with the nine subjects.

In the subsequent section, we showcase the confusion matrices derived from our proposed methodology. Precision, a key metric, delineates the ratio of correct classifications to the total number of tests conducted. Figure 9 elucidates the confusion matrices generated by our proposed model for each individual subject. Each cell within the confusion matrix encapsulates the count of predictions made by the model, accurately categorizing or misclassifying the classes. Notably, correct predictions are aligned along the diagonal of the matrix, whereas mislabeled instances by the classifier manifest off the diagonal. As a fundamental principle, higher values along the diagonal of the confusion matrix signify more precise predictions rendered by the model.

The proposed approach offers several advantages compared to state-of-the-art methods for EEG motor imagery classification. Firstly, the fusion of CNNs allows for the combination of strengths from different CNN architectures, creating a more robust model against noise and better capable of distinguishing between different motor imagery classes. Secondly, the use of simple preprocessing and effective feature extraction has improved classification accuracy. The simple preprocessing reduces noise and normalizes the data. Feature extraction, including frequency features using WPD and spatial features using CSP, captures crucial information in EEG signals related to motor imagery.

While overall system performance is crucial, it is essential to note that issues such as those observed in the case of subject 4, where EEG signals were less distinct and less reliable, result in a decrease in performance. In this specific case, the approach achieved a classification accuracy of only 79.44%. These results underscore the importance of considering the reliability of EEG signals in the continuous evaluation and enhancement of the proposed approach.

## 4. Discussion

This study delves into the intricate task of classifying motor imagination movements in patients diagnosed with motor disability, a domain rife with complexities and challenges. Deep neural networks have garnered significant attention for their potential to process the intricate signals emanating from brain functions. Within this landscape, CNN-based methodologies have emerged as a focal point in research endeavors aimed at classifying IM tasks. However, despite their popularity, it is crucial to critically evaluate their efficacy and limitations.

The comparison of our proposed methodology with other state-of-the-art methods, as referenced in Table 12. The methodologies outlined in [5,20,25,31,32,33,34,35] are assessed utilizing the BCI Competition VI 2a dataset. Notably, our proposed model demonstrates superior performance across all metrics, particularly in terms of accuracy.

The CNN ‘Deeper and Shallow’ in [29] was devised for classifying IM movements using cropped training data, achieving an average accuracy rate of 41.07%. Another method, based on the fusion of features from multi-layer CNNs and compact deep convolutional neural networks (CCNNs), was evaluated using data from BCI Competition VI 2a, achieving average accuracy rates of 75.72% and 73.77%, respectively. In a different approach outlined in [32,36,37,38,39,40,41,42], the authors introduced the spatiotemporal recurrence (STR) and least squares classifier (LSC) as feature extraction techniques and classifiers for multi-class EEG-MI classification, resulting in an accuracy of 49.22%. For the ensemble method [33,43,44,45], the author employed “Adaptive Boosting for Multiclass Classification” (AdBoostM2) as a classifier, with decision trees as the learners, achieving an accuracy of 58.22%.

**Figure 10:**
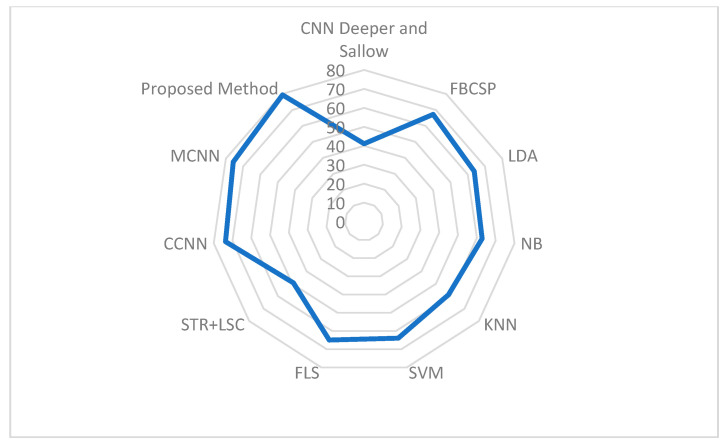
Graphical representation of the comparative results.

Regarding the KNN method [33], K-nearest neighbors (KNNs) were utilized for spot classification. While effective with a sufficiently large number of features, the algorithm’s accuracy can significantly decrease due to noise or irrelevant characteristics, as observed with an accuracy value of 58.88%. In contrast, as depicted in Table 12, our proposed method outperforms all other machine learning methods, achieving an average classification accuracy of 79.44%. Conversely, the STR+LSC method [5] performed the poorest with an average classification accuracy of 58.20%. Additionally, the average classification accuracy of FBCSP is 67.21%, as indicated by the BCI competition results. Figure 10 shows the graphical representation of the comparative results.

Our manuscript embarks on this journey by laying the groundwork with a nuanced exploration of basic physiological concepts, leading to a deeper understanding of the brain’s electrophysiology. We dissect the nervous system, shedding light on the intricate mechanisms underlying cerebral electrical activity. Additionally, we provide insights into the tools employed in brain research, namely EEG and magnetoencephalography (MEG), recognizing their significance in decoding neural signals. MEG offers superior spatiotemporal resolution compared to EEG, thus facilitating the investigation of whether these enhanced signal characteristics result in accelerated BCI transmission speed.

Moreover, we delve into the intricacies of crafting imagery-controlled movements (ICMs), elucidating the neurophysiological phenomena underpinning their creation. This entails an exploration of the requisite skills and knowledge essential for designing robust ICMs based on motor imagination. However, it is imperative to acknowledge the inherent complexities and uncertainties in this process, necessitating a critical lens in interpreting results.

Building upon this foundation, we scrutinize various approaches and existing works in the realm of MI movement classification employing deep learning methodologies. While we advocate for the fusion of multiple CNN architectures in our approach, it is essential to acknowledge the potential drawbacks and challenges associated with such fusion strategies. These may include issues related to model complexity, computational overhead, and interpretability of results [46,47,48].

The results obtained from our fusion strategy indeed demonstrate promising accuracy rates, suggesting the potential efficacy of our approach. However, it is crucial to contextualize these findings within the broader landscape of MI classification methodologies. Comparative analyses with other state-of-the-art methods reveal our approach’s superiority in terms of accuracy. Yet, it is essential to delve deeper into the nuances of performance metrics, considering factors such as robustness, generalizability, and scalability.

This may entail exploring alternative fusion methods, incorporating additional features, and refining preprocessing techniques to mitigate noise and improve signal clarity. Furthermore, a critical examination of the underlying assumptions and limitations of deep learning methodologies in this context is paramount for advancing the field effectively. Limitations of the conducted study are as follows:

- Variations in performance can arise due to several factors, including subject-specific differences in neurophysiological characteristics, electrode placement, and the quality of EEG signals. For instance, some subjects may have higher signal-to-noise ratios, which can lead to more accurate and stable readings, while others may experience artifacts or weaker signals that impact the overall performance;
- Number of subjects taken in the study are limited. It can be enhanced in future research;
- Only some limited methods of CNN are included in the work. Other CNN approaches can be combined for different results;
- This study may not have included all the properties of subjects, which can be further added and may improve the results.

Further performance differences in brain–computer interfaces (BCIs) can be attributed to a range of factors, including inter-subject variability, task-specific nuances, and differences in data processing methodologies. These variations are not merely technical challenges but also provide valuable insights into individual differences in brain functionality and adaptability. From a broader research perspective, understanding and addressing performance differences contributes to the following:

- By recognizing inter-subject variability, researchers can design adaptive algorithms that tailor proposed BCI models to individual users, enhancing usability and reliability.
- Addressing variability pushes the boundaries of machine learning, particularly in areas such as transfer learning and domain adaptation. These techniques are key to developing generalized models that can accommodate diverse users and contexts.
- Investigating the sources of variability offers a deeper understanding of how genetic, cognitive, and environmental factors shape brain activity, enriching the field of neuroscience.
- Performance differences highlight the need for robust and scalable BCI systems capable of functioning effectively in real-world settings, such as assistive technologies and neurorehabilitation.

## 5. Conclusions

When issues with motor skills substantially impede academic performance or day-to-day living tasks, an analysis of motor skills disorder, also called developmental coordination disorder, is made [28,49]. Our proposed model of hybrid five CNNs study makes significant steps in the classification of motor imagination movements by leveraging deep neural networks. Through the fusion of multiple CNN architectures, we have demonstrated promising results in accurately classifying motor imagery EEG signals, surpassing the performance of existing methodologies. The complexities inherent in this domain underscore the need for ongoing refinement and exploration of classification methodologies. Future research should focus on addressing limitations such as model complexity and interpretability while also exploring alternative fusion methods and refining preprocessing techniques.

This study underscores the need for continued exploration and refinement of classification methodologies in MI movement analysis [50]. Future research endeavors should not only seek to enhance classification accuracy but also prioritize robustness, interpretability, and real-world applicability. Some pertinent suggestions for future work are as follows:

- Testing on larger and more diverse datasets collected from a larger number of subjects. This would ensure the generalizability of results and improve the model’s robustness to variations in EEG signals;
- Evaluation on different motor imagery tasks. While the research focuses on motor imagery, it could be applied to other EEG classification tasks such as emotion recognition or attention detection;
- Improving and optimizing the model by fine-tuning the hyperparameters of each CNN, which could potentially enhance the classification accuracy;
- Integrating the approach into a BCI system for practical applications such as prosthetic control or neurorehabilitation. This would require addressing challenges like real-time processing and adaptation to user-specific EEG patterns.

By doing so, we can advance the efficacy and robustness of motor imagery classification systems, ultimately facilitating improved human–machine interaction, assistive technologies, and advancements in neurorehabilitation. This progress is crucial for the medical community, as it aids in the analysis of motor disabilities and supports individuals in managing these conditions effectively [51].

## Figures and Tables

**Table 1:** Comparative analysis of the relative literature.

S. No.	Methodology	Description	Contributions and Limitations
1	LDA [2]	Linear discriminant analysis method for EEG feature extraction	Inadequate knowledge achievedfrom proposed model
2	SVM [3]	Support vector machine classification algorithm for EEG signal analysis	Improved accuracy compared to LDA
3	KNN [4]	k-nearest neighbor algorithm for EEG data classification	Inefficient performance according to different datasets
4	NB [5]	Naïve Bayes algorithm for EEG signal classification	Ineffecient performance in comparison to others
5	Fuzzy Logic System [8]	Utilizes fuzzy logic to classify EEG signals based on power features	Effective for binary classification
6	Wavelet Transform (WT) [9]	Applies WT to extract temporal features for EEG signal classification	Effectivefor binary classification
7	Multioutput Fuzzy Logic System [10]	Incorporates PSO for enhanced classification of multiple-class MI data	Enhanced performance but computationally complex
8	Common Spatial Filter Bank (FBCSP) [11]	Utilizes CSP method for EEG signal analysis	Slightly superior performance compared to baseline
9	CNN [6]	CNNs for extracting discriminative features from EEG signals	Limited improvement over traditional ML techniques
10	CNN + MLP [15]	Fusion of ConvNet with MLP for multi-class imaginary motor task classification	Promising performance for multi-class classification
11	CNN + GB [16]	Combination of CNN with gradient boosting for extracting meaningful information from EEG signals	Effective integration of deep learning and ML approaches
12	CNN-SVM [17]	Combines CNN with SVM for EEG signal analysis	Moderate performance improvement
13	Multibranch 3D CNN [18]	Extracts temporal and spatial features simultaneously from EEG signals	Enhanced feature extraction and classification
14	ConvNet + RNN [22]	Joint utilization of ConvNet and RNN for spatiotemporal feature extraction from EEG signals	Identification of cognitive events
15	MCNN + CCNN [25]	Fusion method of multilayer CNNs for capturing spatial and temporal aspects of EEG data	Outperforms advanced ML and DL techniques
16	SVA+LDA+KNN [26]	Hybrid method of three machine learning methods	Gives effective performance then other previous works
17	Several SVM Methods [27]	Combination of three different support vector machines algortihms	Promising results for specific datasets

**Table 2:** Classification precision obtained by the proposed model.

Subject No.	Precision (%)
1	84
2	75
3	90.7
4	75.2
5	73.7
6	73
7	83
8	85
9	81.5

**Table 3:** Subject 1.

Class	Precision (%)	Recall (%)	F1 (%)
Left-hand	81	79	79.9
Right-hand	88		
Feet	80		
Tongue	87		

**Table 4:** Subject 2.

Class	Precision (%)	Recall (%)	F1 (%)
Left-hand	81	79	79.9
Right-hand	88		
Feet	80		
Tongue	87		

**Table 5:** Subject 3.

Class	Precision (%)	Recall (%)	F1 (%)
Left-hand	81	79	79.9
Right-hand	88		
Feet	80		
Tongue	87		

**Table 6:** Subject 4.

Class	Precision (%)	Recall (%)	F1 (%)
Left-hand	81	79	79.9
Right-hand	88		
Feet	80		
Tongue	87		

**Table 7:** Subject 5.

Class	Precision (%)	Recall (%)	F1 (%)
Left-hand	81	79	79.9
Right-hand	88		
Feet	80		
Tongue	87		

**Table 8:** Subject 6.

Class	Precision (%)	Recall (%)	F1 (%)
Left-hand	81	79	79.9
Right-hand	88		
Feet	80		
Tongue	87		

**Table 9:** Subject 7.

Class	Precision (%)	Recall (%)	F1 (%)
Left-hand	81	79	79.9
Right-hand	88		
Feet	80		
Tongue	87		

**Table 10:** Subject 8.

Class	Precision (%)	Recall (%)	F1 (%)
Left-hand	81	79	79.9
Right-hand	88		
Feet	80		
Tongue	87		

**Table 11:** Subject 9.

Class	Precision (%)	Recall (%)	F1 (%)
Left-hand	81	79	79.9
Right-hand	88		
Feet	80		
Tongue	87		

**Table 12:** Comparative results of our proposed system with state-of-the-art methods.

Methods	Average Accuracy (%)	Reference
CNN Deeper and Sallow	41.07	[30]
FBCSP	67.21	[20]
LDA	63.81	[33]
NB	62.80	[5]
KNN	58.80	[4]
SVM	64.00	[3]
FLS	65.00	[34]
STR+LSC	49.22	[31]
CCNN	73.77	[26]
MCNN	75.72	[26]
Proposed Method	79.44	-

## Data Availability

The corresponding author will provide the information supporting the research study’s conclusions upon reasonable request.
